# Supplementary material for: Evaluation of the Kinetics of Antibody Response to COVID-19 Vaccine in Solid Organ Transplant Recipients: The Prospective Multicenter ORCHESTRA Cohort
Source: Microorganisms. 2022 May 12;10(5):1021. doi: 10.3390/microorganisms10051021 (PMC9147204; doi:10.3390/microorganisms10051021)

Supplemental Table S1. Lower Limits of Quantification (LLQ) and Upper Limits of Quantification (ULQ) for IgG measurements.

	Nucleocapsid	RBD	Spike	Spike (D614G)	Spike (B.1.1.7)	Spike (B.1.351)	Spike (P.1)
LLQ	0.20	0.96	0.79	0.91	0.48	0.27	0.32
ULQ	826	3 944	3 244	3 739	1 982	1 126	1 306

Footer: RBD: rapid binding domain.

Supplemental Table S2: Stratification of antibody response according to WHO criteria

	**Negative**	**Inconclusive**	**Low**	**Medium**	**High**	**Units**
**anti-Spike**	<4.76	4.92 - <53	53 - <241	241 - <832	>832	BAU/mL
**anti-RBD**	<5.58	5.58 - <45	45 - <205	205 - <817	>817	BAU/mL
**anti-N**	<8.20	8.20 - <12	12 - <295	295 - <713	>713	BAU/mL

Footer: WHO: world health organization; RBD: rapid binding domain.

Supplemental Table S3. Number of SOT recipients enrolled at each centre and the distribution of the types of graft per center

	Overall SOT enrolled	Kidney N (% per organ) [% per centre]	Heart N (% per organ) [% per centre]	Liver N (% per organ) [% per centre]	Lung N (% per organ) [% per centre]
Padova	148 (13.94%)	52 (7.68%) [35.14%]	77 (43.5%) [52.03%]	19 (10.44%) [12.84%]	0
Bologna	487 (45.86%)	230 (33.97%) [47.23%]	100 (56.50%) [20.53%]	132 (72.53%) [27.10%]	25 (96.15%) [5.13%]
Verona	97 (9.13%)	69 (10.19%) [71.13%]	0	28 (15.38%) [28.87%]	0
Vicenza	211 (19.87%)	209 (30.87%) [99.05%]	0	1 (0.55%) [0.47%]	1 (3.85%) [0.47%]
Treviso	119 (11.21%)	117 (17.28%) [98.32%]	0	2 (1.10%) [1.68%]	0
Seville	23 (2.12%)	22 (3.15 %) [95.65%]	0	0	1 (3.70 %) [4.35%]

Footer: SOT: Solid organ transplant.

Supplemental Table S4. Positions of Healthcare Workers enrolled.

**Job Title-HCWs**	
Administration	185 (3.69%)
Technician	533 (10.63%)
Nurse	1808 (36.04%)
Physician (including residents)	1390 (27.71%)
Other HCW (including auxiliary workers)	1100 (21.93%)

Footer: HCWs: Healthcare Workers.

Supplemental Figure S1: Study flow-chart

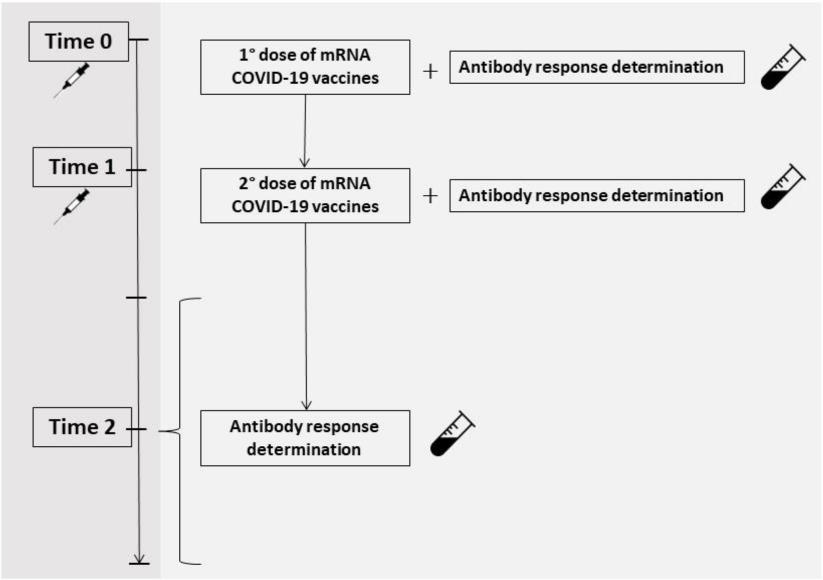

Supplement: Supplementary file 1 [file microorganisms-10-01021-s001.zip › microorganisms-1708906-supplementary.pdf]
